# Translational Concepts of mGluR5 in Synaptic Diseases of the Brain

**DOI:** 10.3389/fphar.2012.00199

**Published:** 2012-11-27

**Authors:** Thomas M. Piers, Dong Hyun Kim, Byeong C. Kim, Philip Regan, Daniel J. Whitcomb, Kwangwook Cho

**Affiliations:** ^1^School of Clinical Sciences, Faculty of Medicine and Dentistry, University of BristolBristol, UK; ^2^Department of Neurology, Chonnam National University Hospital and Medical School, Chonnam National UniversityGwangju, Republic of Korea; ^3^Henry Wellcome Laboratories for Integrative Neuroscience and Endocrinology, MRC Centre for Synaptic Plasticity, University of BristolBristol, UK; ^4^School of Physiology and Pharmacology, University of BristolBristol, UK

**Keywords:** mGluR5, scaffolding proteins, synaptic disease

## Abstract

The G-protein coupled receptor family of glutamate receptors, termed metabotropic glutamate receptors (mGluRs), are implicated in numerous cellular mechanisms ranging from neural development to the processing of cognitive, sensory, and motor information. Over the last decade, multiple mGluR-related signal cascades have been identified at excitatory synapses, indicating their potential roles in various forms of synaptic function and dysfunction. This review highlights recent studies investigating mGluR5, a subtype of group I mGluRs, and its association with a number of developmental, psychiatric, and senile synaptic disorders with respect to associated synaptic proteins, with an emphasis on translational pre-clinical studies targeting mGluR5 in a range of synaptic diseases of the brain.

## Introduction

The metabotropic glutamate receptors (mGluRs), a sub-family of glutamate receptors, are G-protein coupled and share a common molecular morphology with other G-protein-linked receptors. Expression is widespread throughout the mammalian nervous system, including the cerebral cortex (Lopez-Bendito et al., 2002), cerebellar neurons (Berthele et al., 1999), striatal neurons, and the spinal cord (Aronica et al., 2001). Functionally, mGluRs have been implicated as essential mediators of neural development (Zirpel et al., 2000; Di Giorgi Gerevini et al., 2004; Di Giorgi-Gerevini et al., 2005; Jo et al., 2006; Castiglione et al., 2008) and more broadly as important regulators of synaptic strength in the adult brain (Jong et al., 2009; Kumar et al., 2012).

**Figure 1:**
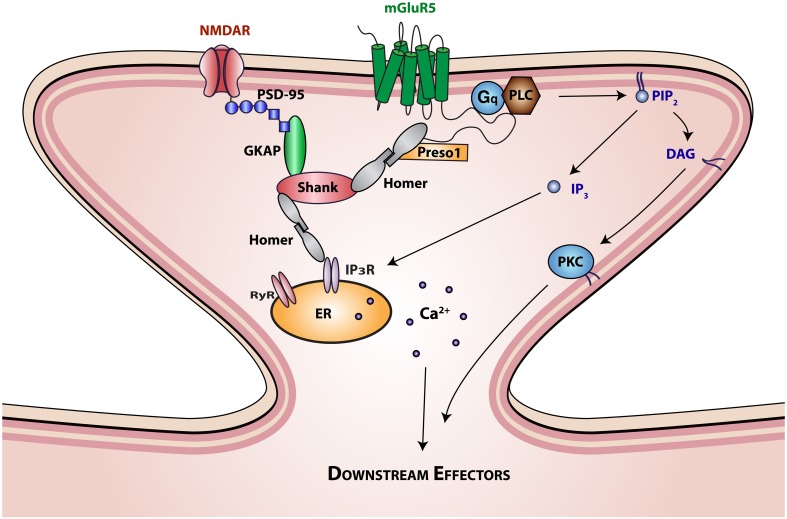
**Interaction of mGluR5 with scaffolding proteins and signaling molecules**. The SH3-multiple ankyrin domain-containing protein (Shank) is a prototypical PDZ scaffolding protein. The PDZ domain of Shank interacts with the c terminus of guanylate kinase-associated protein (GKAP), which is in turn associated with the ionotropic glutamatergic *N*-methyl-D-aspartate (NMDA) receptor-PSD95 complex. The proline rich domain of Shank interacts with the EVH domain of Homer proteins. Homer proteins form multimers through interactions of their coiled-coil domain and link Shank to mGluR5 and inositol triphosphate (IP_3_) or ryanodine receptors. Homer interactions with mGluR5 are further regulated by Preso1 scaffolding proteins. mGluR5 activation by glutamate initiates G_q_ protein signaling that regulates the function of phospholipase C (PLC). Activation of PLC results in the hydrolysis of phosphatidylinositol-4,5-bisphosphate (PIP_2_) to release the second messengers 1,2-Diacylglycerol (DAG) and IP_3_. DAG is the physiological activator of protein kinase C (PKC), which in turn activates various intracellular signaling cascades. IP_3_ binds to intracellular IP_3_ receptors (IP_3_R) on the endoplasmic reticulum (ER) membrane initiating Ca^2+^ release from the ER lumen into the cytoplasm, generating complex Ca^2+^ concentration-dependent signals, including temporal oscillations, and propagating waves.

Metabotropic glutamate receptors can be subdivided into group I (mGluR 1 and 5), group II (mGluR 2 and 3), and group III (mGluR 4, 6, 7, and 8), according to G-protein coupling and mode of signal transduction. Group I receptors, which are expressed in the periphery of the postsynaptic densities of asymmetrical synapses, activate G_q_/G_11_-phospholipase C mediated signaling via inositol phosphate hydrolysis and second messenger production (Figure 1; Berridge and Irvine, 1984). These second messengers are capable of initiating a myriad of cellular responses through their effects on intracellular Ca^2+^ stores and key enzymes, such as protein kinase C (Berridge and Irvine, 1984). In contrast to group I mGluRs, group II, and III mGluRs are distributed presynaptically. These receptors modulate cAMP signaling via the G_i_/G_o_ intracellular pathway (Anwyl, 1992; Shigemoto et al., 1997; Cartmell and Schoepp, 2000).

Group I mGluRs have been a major focus of investigation, particularly mGluR5, which is further subdivided into two structurally different receptor isoforms, named mGluR5a and mGluR5b (Minakami et al., 1993; Joly et al., 1995). mGluR5 is found in almost all brain regions, where through development mGluR5a is the predominant isoform during the early postnatal period, and mGluR5b is more highly expressed in the adult (Minakami et al., 1993; Romano et al., 1996). During development, mGluR5 is present in cells surrounding the lateral ventricle of the embryonic brain, as well as the subventricular zone (SVZ) of the postnatal brain (Di Giorgi Gerevini et al., 2004; Di Giorgi-Gerevini et al., 2005). Here, during these early developmental stages, mGluR5 function is involved in both synaptogenesis and the patterning of neural circuits (Catania et al., 1994; Park et al., 2007; Wijetunge et al., 2008). Importantly, the activity of mGluR5 is influenced by a number of postsynaptic proteins, such as the scaffolding proteins Homer and Shank, which also have significant developmental roles (Tu et al., 1999; Giuffrida et al., 2005; Ronesi et al., 2012).

Given that mGluR5 regulates various mechanisms implicated in neurogenesis and synaptic maintenance, it is interesting to note emerging evidence suggesting that aberrant regulation of mGluR5 leads to mental disorders with strong developmental and synaptic origins. These include Fragile X syndrome (FXS; Dolen and Bear, 2008; Levenga et al., 2011), autism spectrum disorder (Silverman et al., 2010; Mehta et al., 2011; Won et al., 2012), and schizophrenia (Liu et al., 2008; Vardigan et al., 2010; Carlisle et al., 2011). mGluR5 dysregulation in the aged brain is now also emerging as a key mediator of Alzheimer’s disease (AD) pathology, in which synaptic maintenance is strongly impaired (Lee et al., 1995, 2004; Li et al., 2009). Here we shall discuss the role of mGluR5 in synaptic diseases and review the increasing number of candidate drugs targeting the receptor for efficacious treatment in a range of developmental and synaptic diseases.

## mGluR5 and Associated Postsynaptic Proteins

Metabotropic glutamate receptors are tightly regulated by their association with scaffolding proteins. Homer, a member of a postsynaptic family of scaffolding proteins, has been shown to interact with group I mGluRs (Brakeman et al., 1997). Homer proteins exert effects on both the localization and signaling of mGluRs (Thomas, 2002). Specifically, Homer can reduce mGluR5 coupling to postsynaptic effectors, such as the IP_3_ receptor (IP_3_R; Kammermeier and Worley, 2007) and regulate mGluR-mediated protein synthesis (Ronesi and Huber, 2008). Understanding the mechanisms underlying the association between scaffold proteins and mGluRs has now become an area of significant interest. A recent study suggests that mGluR5 regulation by proline-directed kinases, such as cyclin-dependent kinase-5 (cdk-5), leads to enhanced mGluR5-Homer binding. This in turn leads to a reduction in mGluR5-induced cytosolic Ca^2+^ increase, manifesting in a reduction in mGluR coupling to voltage-gated Ca^2+^ channels (Hu et al., 2012). The authors identified that the novel scaffolding protein Preso1 also served to mediate this mechanism; as a multidomain scaffolding protein, Preso1 localized mGluR, Homer and proline-directed kinases, promoting the phosphorylation of mGluR, and subsequent binding of Homer (Hu et al., 2012).

Shank, a family of postsynaptic proteins that function as part of the NMDA receptor (NMDAR)-associated PSD-95 complex, is another mGluR5 regulating scaffolding protein thought critical in mGluR function. Shank clusters mGluR5 and mediates the co-clustering of Homer with PSD-95/GKAP. Thus, Shank may cross-link Homer and PSD-95 complexes in the postsynaptic density and play a role in the signaling mechanisms of both mGluR5 and NMDARs (Tu et al., 1999). Indeed, synergistic interactions between mGluR5 and NMDARs have previously been identified, whereby activation of mGluR5 enhances NMDAR function (Gregory et al., 2011; Won et al., 2012). Additionally, mGluR5 has been shown to drive experience dependent changes in NMDAR subunit composition (Matta et al., 2011). It has also been revealed that an interaction between Shank1B and Homer1b is required for mGluR5-mediated IP_3_ generation and ensuing Ca^2+^ release from intracellular stores (Sala et al., 2005), suggesting a further complexity to the regulation of mGluR5 function by multiple coordinating scaffolding proteins.

## mGluR5 and Synaptic Disease

mGluR5 has been studied as a fine regulator in both embryonic and postnatal neurogenesis (Di Giorgi Gerevini et al., 2004; Di Giorgi-Gerevini et al., 2005; Castiglione et al., 2008). mGluR5 function is therefore critical for synapse formation during brain development, suggesting that dysregulation of mGluR5 signaling might lead to developmental synaptic disorders. Accordingly, the modification of particular mGluR5 scaffolding proteins has been implicated in synaptic diseases and psychiatric disorders (Box 1). It has recently been suggested that dysregulation of Shank could be involved in schizophrenia-induced signaling cascades (Grabrucker et al., 2011). Studies have revealed that two *de novo* mutations in Shank3 are present in a subset of schizophrenia patients (Gauthier et al., 2010) and a Shank1 promoter variant leads to significant working memory deficits in schizophrenia (Durand et al., 2012; Lennertz et al., 2012), suggesting common Shank variants may contribute to neuropsychological dysfunctions in schizophrenia. Furthermore, limited studies also suggest mice lacking mGluR5 show schizophrenia-related behaviors including abnormal locomotor patterns, reduced pre-pulse inhibition (PPI), and deficits on performance of a short-term spatial memory task on the Y-maze (Gray et al., 2009; Wang et al., 2009; Chen et al., 2010), suggesting that positive modulation of mGluR5 function would be a viable target for schizophrenia therapeutics. However, to date, research into the aberrant signaling mechanisms underlying the etiology of schizophrenia have not identified synergistic roles for scaffolding proteins and mGluR5 activity, and further studies are required to elucidate any molecular coupling of these pathways.

Box 1 **Translational concepts through targeting mGluR5.**

Excessive mGluR5 activation has already been alluded to as a potential contributing factor in synaptic disorders, and a number of studies are currently testing the therapeutic potential of drugs that modify mGluR5 signaling through inhibition (Dolen et al., 2010). For these reasons, the development of mGluR5 ligands to combat synaptic diseases has become a highly attractive therapeutic area to pursue (Table 1).

**Autism Spectrum Disorders**

Iossifov et al. (2012) recently described overlap between autism susceptibility genes and the FMR1 gene, involved in FXS, after performing genetic sequencing of autistic children. Among the 59 new autism genes discovered, 14 were associated with fragile X mental retardation protein (FMRP), which is associated with both FXS and regulation of the gene encoding mGluR5 (Sokol et al., 2011). Therefore, given the supposed role of mGluR5 in FXS and autism, the receptor provides an attractive target for drug discovery in these disorders and a number of candidate compounds including mGluR5 negative allosteric modulators (NAMs) have progressed through to Phase II/III clinical trials.

**Epilepsy**

Although no significant clinical trials targeting mGluR5 in epilepsy have so far been performed, group I mGluRs, have been implicated in the disease, a common neurological disorder that occurs more frequently in children than in adulthood (Hauser and Hersdorffer, 1990). Epilepsy is also the most common neurological abnormality in FXS, occurring in approximately 20% of cases, presenting as seizures and EEG abnormalities (Musumeci et al., 1988). Agonists of group I mGluRs act as convulsants (Conn and Pin, 1997), and selective group I mGluR antagonists block seizures in rodent models of epilepsy (Chapman et al., 2000; Yan et al., 2005). It is thought that mGluR1 activation plays a role in sustaining the expression of prolonged bursts, whereas mGluR5 activation may be a contributor to the induction process underlying the epileptogenesis (Stoop et al., 2003). Therefore, blockade of mGluR5 receptors may also be worth exploring as adjunctive strategies for the treatment of seizures.

**Schizophrenia**

There is a growing indication of a specific involvement of mGluR5 in schizophrenia. Recent therapeutic strategies for the treatment of schizophrenia focus on the pharmacological interaction between mGluR5 and NMDA receptors (NMDARs; Homayoun et al., 2004; Stefani and Moghaddam, 2010), as it is well established that mGluR5 synergistically facilitates NMDAR function to alleviate the cognitive deficits associated with schizophrenia (Awad et al., 2000; Attucci et al., 2001; Pisani et al., 2001; Rosenbrock et al., 2010). Moreover, activation of mGluR5 receptors with an agonist or positive allosteric modulator (PAM), such as CDPPB, ADX47273, MPPA, VU0092273, VU0360172, or ADX63365 has shown anti-psychotic-like properties, potentially providing therapeutic efficacy (Conn et al., 2009; Krystal et al., 2010; Niswender and Conn, 2010).

In summary, a number of neurological disorders of developmental origin are promising candidates for mGluR5-targeted therapeutics. Clearly, the balance of mGluR5 activation and inhibition is a critical parameter for healthy formation of neural circuits during development. This concept also extends into the aged brain, where dysregulation of mGluR5 function can have detrimental effects upon synaptic maintenance.

**Table 1:** **mGluR5 modulators in clinical trials**.

Compound	Mechanism	Indication	Clinical T phase	Company
AFQ056	mGluR5 NAM	Fragile X syndrome/PD-LID/Huntington’s chorea/GERD	II/III in fragile X syndrome; II in PD-LID; II in Huntington’s chorea (halted); IIb in GERD (halted)	Novartis
RO4917523	mGluR5 antagonist	Depression/fragile X syndrome	IIa in depression; II in fragile X syndrome	Roche
ADX48621	mGluR5 NAM	PD-LID, focal dystonia	II in PD-LID, focal dystonia	Addex
ADX63365	mGluR5 PAM	Schizophrenia, cognition	Pre-clinical trial in schizophrenia	Addex, Merck and Co
STX107	mGluR5 antagonist	Fragile X syndrome	II in fragile X syndrome	Seaside therapeutics
AZD2516	mGluR5 antagonist	Chronic neuropathic pain/major depression	I/II in healthy volunteers	AstraZeneca
Fenobam	mGluR5 antagonist	Fragile X syndrome/pain	II/pre-clinical in bladder pain	Neuropharm

Deletion of Shank proteins including Shank1 and 2 is believed to produce autism spectrum disorder-like phenotypes (Sato et al., 2012; Won et al., 2012). Recent reports using Shank2 knockout mice, which exhibit an autistic phenotype, showed increased mGluR5 activity and a subsequent decrease in AMPAR synaptic transmission and excessive synapse silencing, further supporting the importance of mGluR5 regulation by scaffolding proteins in synaptogenesis and synaptic disease (Won et al., 2012).

Excessive signaling of mGluR5 is also thought to account for multiple cognitive and syndromic features of FXS, the most common inherited form of mental retardation and autism (Dolen and Bear, 2008). A fragile X mental retardation protein (FMRP) knockout model of FXS induces excessive mGluR5 activation through an increased interaction between mGluR5 and the short Homer isoform, Homer 1a (Ronesi et al., 2012). Furthermore, genetic deletion of Homer 1a, which enhances mGluR5 association with long Homer isoforms, corrected several phenotypes in the FMRP knockout model of FXS, although mGluR-dependent long-term depression (LTD) was not rescued (Ronesi et al., 2012). These findings support the hypothesis that aberrant regulation of mGluR5 can lead to developmental synaptic disorders.

## mGluR5 and Aβ: Convergence in Synaptic Dysfunction

It is widely accepted that certain forms of oligomeric amyloid-beta (Aβ) cause neurotoxicity and contribute to the pathogenesis of AD (Huang and Mucke, 2012). For example, it is now well established that Aβ oligomers, the primary pathology-inducing peptide of AD, can cause synaptic dysfunction, manifesting in the inhibition of long-term potentiation (LTP; Walsh et al., 2002; Shankar et al., 2008; Jo et al., 2011) and the enhancement of LTD (Hsieh et al., 2006; Li et al., 2009). These two forms of synaptic plasticity, exhibited in response to differential neural activity patterns, are believed by many to be the cellular mechanism of learning and memory, the loss of which is a major symptom in AD (Shankar et al., 2008).

Interestingly, it is now known that mGluR5 plays an important role in synaptic plasticity. This is the result of extensive research dedicated to elucidating the involvement of mGluR5 in LTP (Bashir et al., 1993; Bortolotto and Collingridge, 1993) and LTD (Oliet et al., 1997; Bellone et al., 2008). In line with this, recent data confirms positive modulation of mGluR5 induces an enhancement of learning and memory in the normal state as well as in the NMDAR dysfunction-induced amnesic state (Homayoun and Moghaddam, 2010; Rosenbrock et al., 2010; Stefani and Moghaddam, 2010; Menard and Quirion, 2012).

Although limited data directly associates mGluR5 signaling with AD, studies have shown potential mechanistic interactions between AD-associated molecules and mGluR5. Groups focused on molecular pathways in Autism and FXS suggest that activation of mGluR5 removes the repressive effect of the FMRP on amyloid precursor protein (APP) mRNA translation and stimulates secretion of APP (Sokol et al., 2011), the precursor to Aβ peptide generation. Inhibitors of mGluR5 have been shown to reduce Aβ production and to have positive effects upon disease phenotypes in rodent models of FXS and AD (Malter et al., 2010). Together, this suggests aberrant mGluR5 activation may positively regulate the processing of Aβ oligomers leading to disease pathology.

Recent translational studies have also shown that Aβ-mediated impairment of LTP can be attenuated by co-treatment with the mGluR5 antagonist, MPEP (Wang et al., 2004; Rammes et al., 2011), suggesting mGluR5 may be an efficacious target for the treatment of AD. However, although MPEP is characterized as an effective mGluR5 antagonist, a non-specific action as a non-competitive NMDAR antagonist could potentially provide the observed neuroprotective effects (O’Leary et al., 2000).

Evidence for a direct mechanistic interaction between mGluR5 and Aβ was identified by Renner et al. (2010) using quantum dot tagged oligomers of Aβ to show that clustering of Aβ at the membrane greatly reduced the ability of mGluR5 to laterally diffuse from the synapse, resulting in facilitation of mGluR5-mediated signaling. Additionally, Aβ binding to the neuronal surface of hippocampal cultured neurons from mGluR5 knockout mice was greatly reduced, suggesting that mGluR5 may play a reciprocal role in “scaffolding” Aβ oligomer clusters to the synapses. Together these findings hint at a synergistic interaction between Aβ and mGluR5, resulting in the over-activation of mGluR5 with subsequent pathological effects upon NMDAR function and Ca^2+^ homeostasis (Renner et al., 2010).

Finally, Aβ-mediated activation of mGluR5 may inhibit the function of muscarinic acetylcholine receptors (mAChR) leading to cholinergic hypofunction, a hallmark of AD pathogenesis closely linked to Aβ and Tau neuropathologies (Fisher, 2012). Indeed, aberrant expression of mGluR5 leads to the inhibition of mAChR-mediated long-term synaptic depression in the perirhinal cortex (Jo et al., 2006) and acute nicotine-mediated enhancement of LTP in the rat dentate gyrus can be inhibited by MPEP (Welsby et al., 2006). Thus, mGluR5 dysregulation has been proposed as a key step in AD pathology, marking mGluR5 as a possible target to tackle synaptic dysfunction in AD. However, more effort to shed light on the mechanism of action of mGluR5 will be required prior to the effective development of receptor-based treatments for AD.

## Concluding Remarks

In the past several years, significant progress has been made into the understanding of mGluR5 receptor function in health and disease. Studies suggest that the mGluR5 receptor has an important role in synaptogenesis and the regulation of synaptic plasticity. Thus, it seems aberrant mGluR5 regulation and signaling may play a part in a number of synaptic disorders, including a novel role in AD. Therapeutic treatments are slowly coming to the fore in many of these disorders. However, more research into the underlying involvement of this promiscuous receptor in synaptic-related disorders will ensure more efficacious treatments can be developed in the future.

## Conflict of Interest Statement

The authors declare that the research was conducted in the absence of any commercial or financial relationships that could be construed as a potential conflict of interest.
